# Seasonal Patterns of Body Temperature Daily Rhythms in Group-Living Cape Ground Squirrels *Xerus inauris*

**DOI:** 10.1371/journal.pone.0036053

**Published:** 2012-04-27

**Authors:** Michael Scantlebury, Marine Danek-Gontard, Philip W. Bateman, Nigel C. Bennett, Mary-Beth Manjerovic, Kenneth E. Joubert, Jane M. Waterman

**Affiliations:** 1 Mammal Research Institute, Department of Zoology and Entomology, University of Pretoria, Pretoria, South Africa; 2 School of Biological Sciences, Queen's University Belfast, Belfast, Northern Ireland, United Kingdom; 3 Department of Biology, University of Central Florida, Orlando, Florida, United States of America; 4 Section Pharmacology, Department of Paraclinical Sciences, Faculty of Veterinary Science, University of Pretoria, Pretoria, South Africa; 5 Department of Biological Sciences, University of Manitoba, Winnipeg, Manitoba, Canada; University of Plymouth, United Kingdom

## Abstract

Organisms respond to cyclical environmental conditions by entraining their endogenous biological rhythms. Such physiological responses are expected to be substantial for species inhabiting arid environments which incur large variations in daily and seasonal ambient temperature (T_a_). We measured core body temperature (T_b_) daily rhythms of Cape ground squirrels *Xerus inauris* inhabiting an area of Kalahari grassland for six months from the Austral winter through to the summer. Squirrels inhabited two different areas: an exposed flood plain and a nearby wooded, shady area, and occurred in different social group sizes, defined by the number of individuals that shared a sleeping burrow. Of a suite of environmental variables measured, maximal daily T_a_ provided the greatest explanatory power for mean T_b_ whereas sunrise had greatest power for T_b_ acrophase. There were significant changes in mean T_b_ and T_b_ acrophase over time with mean T_b_ increasing and T_b_ acrophase becoming earlier as the season progressed. Squirrels also emerged from their burrows earlier and returned to them later over the measurement period. Greater increases in T_b_, sometimes in excess of 5°C, were noted during the first hour post emergence, after which T_b_ remained relatively constant. This is consistent with observations that squirrels entered their burrows during the day to ‘offload’ heat. In addition, greater T_b_ amplitude values were noted in individuals inhabiting the flood plain compared with the woodland suggesting that squirrels dealt with increased environmental variability by attempting to reduce their T_a_-T_b_ gradient. Finally, there were significant effects of age and group size on T_b_ with a lower and less variable T_b_ in younger individuals and those from larger group sizes. These data indicate that Cape ground squirrels have a labile T_b_ which is sensitive to a number of abiotic and biotic factors and which enables them to be active in a harsh and variable environment.

## Introduction

Organisms respond to cyclical variation in environmental conditions by entraining their endogenous biological rhythms [1], [2]. One such rhythm in endothermic species is that of body temperature (T_b_), which is considered to be a consequence of the balance between heat production and heat dissipation [3]. In many taxa, T_b_ daily rhythms are influenced by diel and seasonal changes in photoperiod and ambient temperature (T_a_) [4]–[9]. Indeed, the primary cues for seasonal acclimatization of the thermoregulatory system, which include changes in T_b_ daily rhythms, are photoperiod and temperature [10], [11]. Interestingly, little is known about which selective pressures may affect the evolution of heterothermy in endotherms. Indeed, it is unclear whether one should examine the effects of environmental variation on raw T_b_ data or use some index which can be comparable across species (e.g. ‘Heterothermy Index’, ‘HI’ [12]). Angilletta et al. (2010) [13] suggest that future empirical work should examine the potential “selective pressures imposed by regional and temporal heterothermy”. They identify several potential candidates which might cause T_b_ variations to evolve which include food and water availability, T_a_ and social huddling. For example, restricted food and water supplies and low T_a_ values should favor energy-saving reductions in T_b_ and temporal heterothermy. Implicit in their arguments is the fact that extremes of variation in T_a_ and in particular cyclical variations in T_a_ may result in adaptive variation in T_b_ daily rhythms [13]–[16]. For group-living animals, behaviors such as social huddling may be one mechanism to conserve water and energy [17], [18]. Minimization of thermoregulatory costs and water loss are thus seen as a possible selective pressure for aggregation [19]–[21]. For instance, huddling in newborn rabbit (*Oryctolagus cuniculus*) pups not only saves energy but also affects T_b_ daily rhythms [22]. Hence, T_b_ daily rhythms are likely to be affected by group size in social animals.

The open thorn scrub savannah ecosystem of southern Africa is subject to wide diel and annual variations in temperature across seasons, often reaching above 40°C during the summer and below freezing during the winter [23]. In this habitat, large open areas are interspersed with occasional stands of trees and bushes that generally concentrate in depressions around pans and dry river beds [24]. These areas are likely to present different microclimatic conditions due in part to differences in exposure to solar radiation [25]. Small mammals that inhabit this region, such as the Cape ground squirrel (*Xerus inauris*), exhibit typical arid adaptations including a low resting metabolic rate, a high thermal conductance and a concentrated urine [26], [27]. They are active year-round and forage during the heat of the day. It has been suggested that they use both behavioral and physiological means to deal with the extremes of T_a_ they encounter [28]–[30]. For example, they may be active during hot summer days because they periodically dissipate body heat by retreating to cooler burrows [31]. Therefore, it is likely that their T_b_ will vary considerably, both on a daily and a yearly basis, as a physiological adaptation to reduce the T_a_-T_b_ gradient [5], [32], [33]. However, it is unknown how this is related to microhabitat and behavior, such as the time animals emerge in the morning and how they may interact socially with one another.

Here we investigated the role of T_b_ daily rhythms as a response to seasonal and diel changes in T_a_ in Cape ground squirrels that inhabit a habitat mosaic exposed to large daily and annual temperature fluctuations. Our hypotheses were related to the middle (mesor); the amplitude and the acrophase (time of the peak) of T_b_ daily rhythms [34]. We predicted that: (a) seasonal differences in T_b_ daily rhythms would be apparent with higher mesor values and later acrophase times during the spring and summer; (b) rapid changes in T_b_ would be apparent in the early mornings (after emergence) and a T_b_ would be maintained at a constant level throughout the daylight hours because animals will move into and out of cooler locations such as their burrows as part of their thermoregulatory behavior; (c) lower mesor and amplitude values of T_b_ would be observed in a shaded compared with an open habitat; and (d) winter mesor values would be higher in animals from larger group sizes because of the thermoregulatory benefits gained from huddling at night. In addition, we examined the potential seasonal variation in HI values from individuals inhabiting different locations and from different group sizes to gauge whether or not relationships that emerge when analyzing T_b_ data are also manifest when using this index.

## Materials and Methods

### Ethics statement

Permission was granted from South Africa Northwest Parks and Tourism to conduct the field research. The protocol was approved by committee on the ethics of animal experiments of the Universities of Central Florida and Pretoria (permit number UCF IACUC #07-43W). The study was performed in accordance with the recommendations in the Guide for the Care and Use of Laboratory Animals of the National Institutes of Health.

### Animals and study site

Cape ground squirrels are small (∼600 g), non-hibernating, diurnal, social rodents that inhabit arid regions of sub-Saharan Africa [35]–[37]. They are cooperative breeders with low reproductive skew and a high operational sex ratio. Groups typically consist of 1–6 related females and their sub adult and juvenile offspring, which share a burrow cluster [35], [38]. The study took place at S. A. Lombard Nature Reserve (3,660 ha, 18 km north west of Bloemhof, South Africa, 27°35′S, 25°23′E) as part of an on-going study where squirrels have been studied since 2002. The site comprises *Cymbopogon-Themeda* veld and Kalahari grasslands, and is situated on a flood plain [24]. Mean annual precipitation is 500 mm [39]. Animals were trapped from groups at two locations: an open unshaded area – “the flood plain” – and a habitat containing *Acacia karoo and A. erioloba* stands – “the woodland”, which was approximately 2 km away [40]. Tomahawk wire-mesh traps (15×15×50 cm) baited with peanut butter were used to catch animals, after which they were freeze-marked for unique identification (Quick Freeze, Miller-Stephenson Chemical Co., Danbury, CT [41]) and implanted with transponders (PIT tags, AVID Inc., Norco, CA). The sides of animals were also painted with various shapes using black hair dye (Rodol D, Lowenstein & Sons Inc., New York, NY) so their identities could be seen at a distance. Body mass was recorded along with the size of the social groups to which animals belonged. Trapping took place for two one-week periods during May and October. Age was assessed by knowing dates of first emergence from the natal burrow [35], [42]. Behavioral observations, including times of emergence and immergence from burrows were obtained as outlined in Waterman [37]. Briefly, this involved recording time budgets of individual animals by focal sampling in which all-occurrence data were recorded for periods of up to 20 minutes whereas the activities of all the individuals within a group were recorded every five minutes by scan sampling [43]. We were interested in many different aspects, but in particular movement and foraging activities as well as aggressive, reproductive and social/dominance interactions between individuals.

### Acquisition of body temperature (T_b_) data

Ten squirrels (five sub adults and five adults) were obtained from the flood plain and 10 (also five adults and five sub adults) from the woodland. Sub adults are defined as animals between six months after first emergence from the natal burrow and sexual maturity (around eight months for males and nine months for females); adults are individuals which have reached sexual maturity [38]. Miniature temperature recording iButton® dataloggers (DS1922L±0.0625°C; Thermochron, Dallas Semiconductors, Maxim Integrated Products, Inc., Sunnyvale, CA) were surgically implanted into the peritoneal cavity of each individual under anaesthesia (see below). Prior to surgery, devices were calibrated using an APPA 51 digital thermometer in a water bath. They were set to record every 60 min providing 23 weeks of continuous recordings. Dataloggers were then coated with medical grade surgical wax (ELVAX) [44] and sterilized with formaldehyde vapor. Measurements of T_b_ were recorded between May 17^th^ and October 28^th^ 2006.

Squirrels were anaesthetized with medetomidine (Domitor, Pfizer Laboratories (PTY) Ltd, Sandton) (67.6±9.2 µg/kg), ketamine (Anaket V, Centaur Laboratories (PTY) Ltd, Isando) (13.6±1.9 mg/kg) and buprenorphine (Temgesic, Ricketts Laboratories, Isando) (0.5±0.06 µg/kg) [45]. Anesthesia was induced after 3.1±1.4 minutes. The abdomen was surgically prepared with a chlorhexidine scrub (Hibiscrub, ICL Laboratories), then with chlorhexidine and alcohol (Hibitane, ICI Laboratories). A midline celiotomy was performed for insertion of the dataloggers. The linea alba was closed with 4/0 polydioxanone (PDS, Ethicon, Midrand) and the skin was closed with an intercuticular suture pattern with 4/0 polydioxanone. The procedure for each individual lasted approximately 20 minutes. At the end of the surgical procedure, anesthesia was reversed with atipamezole (Antisedan, Pfizer Laboratories) (232±92 µg/kg). Recovery occurred within 3.5±2.2 minutes. This procedure was followed for removal of dataloggers for the case of five animals that were recaptured. Three other recaptured animals were euthanized with an overdose of halothane upon recapture as part of a different study [46]. Only eight of the total 20 animals implanted were recaptured. After removal of dataloggers, T_b_ data were downloaded using iButton®-TMEX software version 3.21 (2004 Dallas Semiconductor MAXIM Corporation). All animals were observed overnight after implantation and removal of dataloggers and returned to their capture site the following morning. No animal died due to surgical procedures during this period.

### Ambient temperature and daylight measurements

Ambient air temperature (T_a_) was determined using two methods. We set dataloggers to record every hour for the first 84 days (12 weeks) of the sampling period. One datalogger was used per study site. Dataloggers were placed inside Stevenson screens located 90 cm above the ground. To obtain data over a longer time period, we used daily minimum, maximum and mean ambient temperatures recorded at Bloemhof 27.65 S, 25.60 E, GMT +2 (South African Weather Bureau, Pretoria) for the entire 23 weeks of the sampling period; mean hours of sunlight as well as the times of sunrise (civil dawn) and sunset (civil dusk) were also noted. In an attempt to measure underground temperatures, we also placed two dataloggers inside what we thought were disused squirrel burrows. However, these devices did not provide useful information because the burrows were not vacant; squirrels removed them from the burrows and they were found in spoil heaps on the surface.

### Data analyses

Cosinor analysis was used to determine the T_b_ daily rhythms of the individuals measured [34], [47]. The mean mesor, amplitude and acrophase values of the T_b_ daily rhythms were calculated for every individual for each of the 23 weeks of the study period (‘T_b_mesor’, ‘T_b_amplitude’ and ‘T_b_acrophase’, respectively). The significances of the fitted curves were tested against the null hypothesis that the amplitude was zero [48]. The variability in the data that could be accounted for by the fitted curve (percentage rhythm) was calculated. In addition, we calculated the HI values for each animal for each week of the study and assessed whether there were any relationships between HI and season, age or group size. Statistical analyses were performed using SPSS 17 (SPSS Inc., Chicago, IL, U.S.A.). Mean values are reported ± standard deviations.

#### (1) Seasonal variation in Tb daily rhythms

Linear mixed models were used to examine the variation in T_b_ cosinor parameters (mesor, amplitude, acrophase) as a function of time (over the 23 week period). Each dependent variable was analyzed separately. ‘Individual ID’ was included as a random factor to avoid pseudoreplication and to correct for repeated measurements. ‘Week’ was included as fixed covariate. As several explanatory terms and their interactions were investigated, models were selected in a stepwise backward fashion, removing the least significant explanatory terms until the most parsimonious model was obtained, determined by Akaike's information criterion (AIC). Interaction terms were only included when they were significant.

#### (2) Effect of light and ambient temperature (Ta) on body temperature (Tb) daily rhythms

Linear Mixed Models were used to examine the effects of light and T_a_ on the mean weekly cosinor parameters. First, we obtained several measures of T_a_: the daily minimum (T_a_min), the daily maximum (T_a_max) and the daily mean value (T_a_mean) (South African Weather Bureau). We then calculated weekly averages of T_a_min, T_a_max and T_a_mean and included each of these in a model with individual identity as a random factor and week as a fixed effect. This corrected for repeated measurements and differences in mean values between individuals. All potential interactions between temperature variables were included. Models were selected by removing the least significant explanatory terms sequentially until the most parsimonious model was obtained using AIC. Each dependent cosinor variable was analyzed separately. Second, we assessed the effects of various ‘light’ variables on the cosinor variable. The light variables we used were: the weekly average time of sunrise, the weekly average time of sunset and the weekly average length of the photophase. As before, models were selected using AIC by removing least significant explanatory terms sequentially. Finally, for each of the dependent cosinor variables, combined models were undertaken which included the factors with most explanatory power from both the individual T_a_ models and the individual light models. Again, for each analysis the best model was obtained using AIC.

#### (3) Relationship between emergence and immergence times and Tb daily rhythms

Emergence and immergence times for the two habitats were calculated as the mean observed emergence and immergence time of groups of squirrels inhabiting both areas [35]. Data were collected over seven months of detailed observation time recording when individual squirrel groups from the two habitats emerged or immerged. An average of 8.1±0.65 squirrels from different groups were observed every week to calculate emergence times and 5.7±0.81 squirrels from different groups were observed every week to calculate immergence times. Temporal variation in mean emergence and immergence times was investigated using linear regressions. In order to determine how daily variations in T_b_ were related to the times of emergence and whether this differed throughout the year, we computed, for each day, the mean T_b_ of each individual one hour before the time of emergence and the mean T_b_ one hour after the time of emergence. The difference in T_b_ between these two values was then calculated as a percent of the maximum amplitude difference in T_b_ for that individual for that day. The mean percent T_b_ change for each individual was then calculated for each week, after which the mean change for all individuals was calculated for the 23 weeks.

#### (4) Effect of habitat on Ta and Tb daily rhythms

To examine whether mean daily T_a_ differed between the flood plain and the woodland we conducted linear mixed models with habitat as a fixed factor, week as time and T_a_ measured at both study sites as the dependent variable. To determine whether high values of T_a_ obtained during the day or low values obtained during the night differed between the two habitats we included day/night as an additional fixed factor. The hourly T_a_ obtained at both study sites were considered as being ‘daytime’ T_a_ if the measurement was taken between the sunrise and sunset of a given day, and ‘night-time’ T_a_ if the measure was taken between sunset and sunrise time between two consecutive days. An average T_a_ was then determined for each daytime and each night-time period for the 84 days (12 weeks) of the sampling period. To examine the effect of habitat on mean weekly T_b_ values and cosinor parameters, we included ‘habitat’ and ‘day/night’ as a fixed factors, ‘individual’ as random variable and ‘week’ as factor.

#### (5) Effect of age and group size on Tb daily rhythms

Effects of age and group size on T_b_mean, T_b_mesor, T_b_amplitude, T_b_acrophase and HI were conducted using linear mixed models with ‘individual’ as a random variable and ‘week’ as factor. Models were selected in a stepwise manner using AIC as described previously. Age (adult/sub adult) was included as a categorical factor and group size as a continuous variable.

## Results

**Figure 1:**
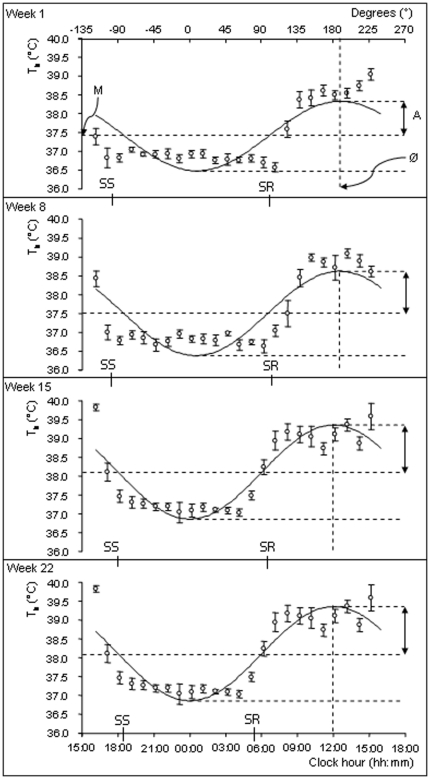
Body temperature (T_b_) daily rhythm of an adult Cape ground squirrel (605 g) for the first (21 to 28 May), eighth (09 to 16 July), fifteenth (27 August to 03 September) and twenty-second week (15 to 22 October) of a 23-week measurement period. ‘M’ indicates the mesor (37.41°C), ‘A’ the amplitude (0.92°C) and ‘Ø’ the acrophase (189.11° or 12∶36 h) of the fitted cosine curve. SR and SS show times of sunrise and sunset.

Of the 20 individuals originally implanted with dataloggers, eight were recaptured; six from the flood plain (two adults, four sub adults) and two from the woodland (two adults). Group sizes (i.e. the sizes of groups in which the eight animals lived) ranged from one to nine individuals. The implanted animals were regularly observed during the two weeks following implantation and no mortality or immigration was observed. We observed no signs of different behavior of the implanted squirrels compared to the others. There were significant daily rhythms of T_b_ in all of the eight individuals measured (Table 1, Fig. 1) with mean ±SD values of the mesor, amplitude and acrophase for the 23 week measurement period of 37.51±0.15°C, 1.13±0.08°C and 12∶33±2 min, respectively.

**Table 1:** Mean (±SE) of the mesor (°C), amplitude (°C), acrophase (time hh:mm) and percentage rythmicity obtained from 24 h cosine functions of hourly T_b_ recordings of eight Cape ground squirrels during a 23-week sampling period.

Week	Begin date	End date	Mesor	Amplitude	Acrophase hh:mm	Percentage rythmicity
1	21/05/2006	28/05/2006	37.37 (0.12)	0.93 (0.08)	12:55 (0:11)	50.32 (4.27)
2	28/05/2006	04/06/2006	37.47 (0.10)	1.16 (0.10)	12:36 (0:05)	58.84 (4.74)
3	04/06/2006	11/06/2006	37.36 (0.08)	1.22 (0.09)	12:37 (0:04)	64.63 (2.80)
4	11/06/2006	18/06/2006	37.35 (0.11)	1.11 (0.10)	12:36 (0:12)	56.12 (4.94)
5	18/06/2006	25/06/2006	37.38 (0.09)	1.07 (0.11)	13:01 (0:16)	53.71 (4.66)
6	25/06/2006	02/07/2006	37.28 (0.09)	1.07 (0.13)	12:31 (0:10)	46.42 (4.19)
7	02/07/2006	09/07/2006	37.42 (0.09)	1.18 (0.12)	12:41 (0:10)	62.49 (3.38)
8	09/07/2006	16/07/2006	37.48 (0.12)	1.10 (0.10)	12:40 (0:05)	60.63 (3.86)
9	16/07/2006	23/07/2006	37.46 (0.12)	1.13 (0.10)	12:43 (0:11)	64.48 (2.89)
10	23/07/2006	30/07/2006	37.51 (0.12)	1.22 (0.07)	12:39 (0:05)	68.00 (1.78)
11	30/07/2006	06/08/2006	37.27 (0.14)	1.00 (0.07)	12:43 (0:06)	51.43 (3.55)
12	06/08/2006	13/08/2006	37.46 (0.11)	1.21 (0.09)	12:32 (0:06)	63.94 (3.24)
13	13/08/2006	20/08/2006	37.55 (0.11)	1.22 (0.07)	12:31 (0:09)	65.30 (1.58)
14	20/08/2006	27/08/2006	37.44 (0.12)	1.04 (0.10)	12:38 (0:11)	54.78 (4.70)
15	27/08/2006	03/09/2006	37.55 (0.14)	1.16 (0.09)	12:40 (0:09)	56.88 (3.80)
16	03/09/2006	10/09/2006	37.79 (0.11)	1.27 (0.08)	12:23 (0:05)	69.15 (1.85)
17	10/09/2006	17/09/2006	37.76 (0.12)	1.20 (0.08)	12:29 (0:06)	68.12 (1.75)
18	17/09/2006	24/09/2006	37.72 (0.12)	1.22 (0.09)	12:19 (0:08)	67.99 (2.19)
19	24/09/2006	01/10/2006	37.68 (0.15)	1.09 (0.08)	12:21 (0:07)	59.17 (3.02)
20	01/10/2006	08/10/2006	37.61 (0.14)	1.12 (0.09)	12:13 (0:05)	65.07 (2.88)
21	08/10/2006	15/10/2006	37.61 (0.15)	1.09 (0.06)	12:15 (0:05)	61.46 (2.74)
22	15/10/2006	22/10/2006	37.59 (0.14)	1.15 (0.06)	12:27 (0:08)	63.16 (2.33)
23	22/10/2006	28/10/2006	37.65 (0.12)	1.06 (0.07)	12:17 (0:10)	50.39 (4.56)
Mean	21/05/2006	28/10/2006	37.51 (0.03)	1.13 (0.02)	12:33 (0:02)	60.11 (1.34)

### (1) Seasonal variation in T_b_ daily rhythms

**Figure 2:**
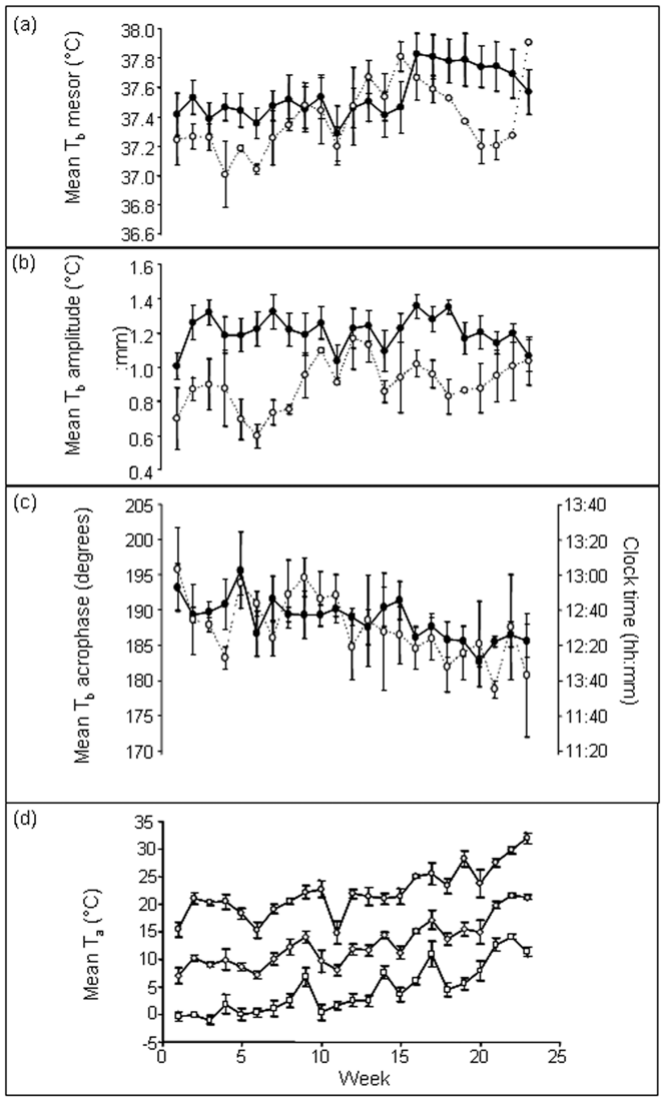
Mean ±SE daily rhythm parameters of eight Cape ground squirrels during the 23 week measurement period for: (a) T_b_ Mesor (°C); (b) T_b_ Amplitude (°C); (c) T_b_ Acrophase (time of day and degrees). Individuals inhabiting the flood plain and the woodland are denoted by solid and open circles. Maximum, minimum and mean T_a_ values are shown in (**d**) as top, middle and lower lines.

There were significant effects of both ‘week’ and ‘individual’ on T_b_mesor and T_b_acrophase (F_1,175_ = 35.86, p<0.001 and F_7,175_ = 8.51, p<0.001 respectively; Fig. 2A, 2C) indicating that mesor values increased significantly and acrophase values became earlier over the time period, and that these values differed between individuals. There was also a significant interaction between individual and week on T_b_amplitude (F_7,168_ = 2.60, p<0.05; Fig. 2B) indicating that changes in amplitude differed between individuals over time.

### (2) Effect of light and T_a_ on T_b_ daily rhythms

Mean T_a_ values ranged from 7.0±1.4°C during the first week to 21.1±0.43°C during the last with daily minimum and maximum values of −3°C and 22°C, and 9°C and 36°C respectively (Fig. 2D). By comparison, mean T_b_ ranged from 37.37±0.11°C during the first week to 37.70±0.12°C during the last. This corresponded to minimum and maximum T_b_ values of 34.28 and 40.11°C, and 35.64°C and 41.23°C, respectively (Fig. 2A).

When the effects of ambient conditions on T_b_ were examined the only ‘temperature’ variable (of T_a_min, T_a_mean and T_a_max) that significantly influenced T_b_mesor was T_a_max (F_1,60_ = 23.87, p<0.001). Similarly, the only ‘light’ variable that significantly affected T_b_mesor was the time of sunset (F_1,99_ = 23.72, p<0.001). When both explanatory terms were included into the same model, neither had a significant effect (p>0.1 in both cases). In contrast, although T_a_max had a significant effect on T_b_amplitude (F_1,53_ = 12.43, p<0.01), T_a_mean and sunrise were the factors that significantly affected T_b_acrophase (F_1,64_ = 29.80, p<0.001 and F_1,78_ = 42.05, p<0.001 respectively), with sunrise being the most important factor in the combined model (F_1,45_ = 10.90, p<0.01).

### (3) Relationships between emergence and immergence times and T_b_ daily rhythms

**Figure 3:**
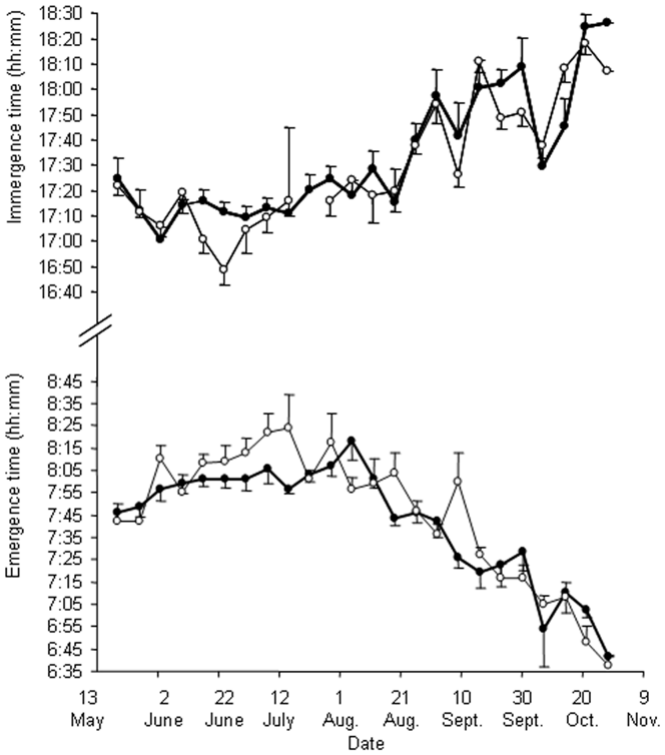
Mean ±SE immergence and emergence times in the flood plain (solid circles and bold line) and woodland (open circles and light line). Mean number of animals observed at any one time was 8.1±4.5 at emergence and 5.6±2.6 at immergence.

**Figure 4:**
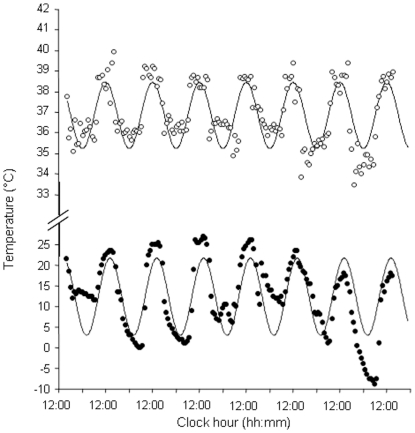
T_b_ (open circles) and T_a_ (solid circles) and fitted cosine curves for a Cape ground squirrel during the 9^th^ week of the sampling period illustrating the variation in T_a_ and T_b_. The difference between the lowest T_b_ value recorded (33.39°C at 19:08) and the highest T_b_ during the previous day (39.32°C at 16:08) was 5.93°C. Over the 23 week period, extreme changes in T_b_ included one individual that decreased in T_b_ by 5.56°C and another that increased in T_b_ by 5.98°C in one hour.

**Figure 5:**
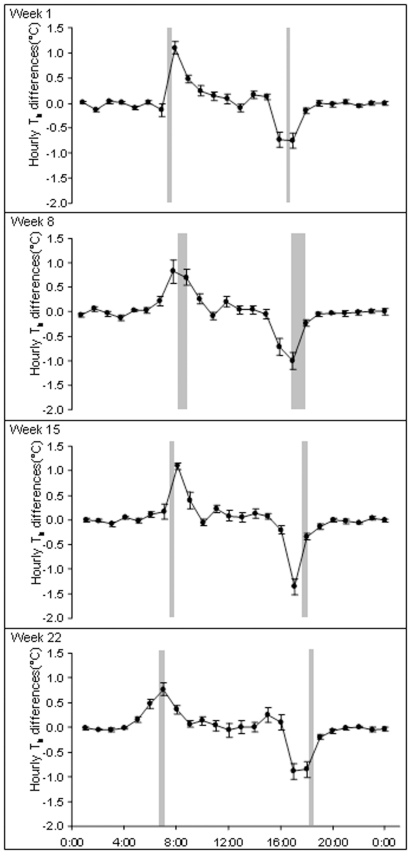
Mean ±SE T_b_ changes between successive hours across all eight individuals during the first, eighth, fifteenth and twenty-second weeks of the measurement period. Grey bars represent the mean ±SE times of emergence (left-hand bar) and immergence (right-hand bar).

Animals emerged later in the day at the beginning of the measurement period (07∶44) (May), than at the end (October) (06∶40) (least-squares regression, F_1,46_ = 63.25, r^2^ = 0.579, p<0.001). In contrast, immergence times occurred earlier in the day at the beginning of the measurement period (17∶24) than at the end (18∶17) (F_1,45_ = 103.02, r^2^ = 0.696, p<0.001)(Fig. 3). There were no differences in emergence and immergence times between animals that inhabited the flood plain and the woodland (emergence: F_1,46_ = 0.19, p = 0.662; immergence: F_1,45_ = 0.17, p = 0.685). However, there was an indication that variation in T_b_ on a day-by-day basis reflected variation in T_a_ with depressions in T_b_ occurring at similar times to depressions in T_a_ (Fig. 4). Changes in T_b_ over 24 h periods were greatest at around the times of emergence and immergence, sometimes in excess of 5°C, highlighting the potential relationship between T_b_ and whether or not the animals were above or below ground (Fig. 5). During the winter (week 1), mean increases in T_b_ for the hour following emergence were +1.10±0.12°C, which were greater than changes in T_b_ which occurred in the hour preceding emergence of −0.14±0.13°C. During the end of the measurement period at week 22, increases in T_b_ following emergence were less at +0.77±0.12°C compared to +0.48±0.10°C during the hour prior to emergence, respectively. There was a significant difference in the T_b_ increase between the beginning and the end of the measurement period, with a 52% increase in T_b_ during the first hour following emergence (relative to the total change in T_b_ during that day) during week one and only a corresponding 20% increase in T_b_ during week 22 (F_1,20_ = 4.99, r^2^ = 0.20, p<0.05). T_b_ values stabilized when animals returned to their burrows in the evening; changes in T_b_ of −0.01±0.06°C were recorded during the hour post immergence and −0.16±0.06°C during the hour prior to immergence for week 1; this compared to changes of −0.08±0.04°C and −0.20±0.04°C, for post-and pre-immergence times during week 22, respectively.

**Figure 6:**
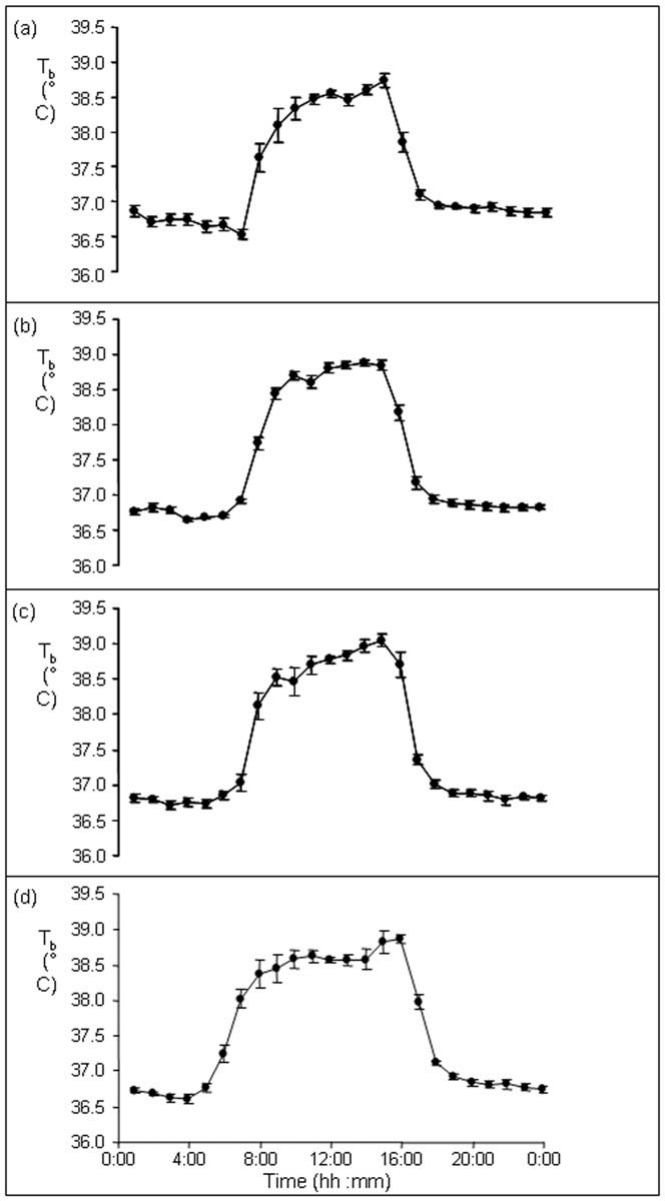
Mean ±SE T_b_ of the eight individuals for the first, eighth, fifteenth and twenty-second weeks of the sampling period. T_b_ values rose rapidly in the morning before reaching a plateau during the day.

The mean time at which T_b_ began to decrease in the mornings across all seasons was 10:13±0:19 minutes and 38.70±0.06°C (Fig. 6). This time became earlier as the measurement period progressed from week 1 to week 22. For the weeks 1, 8, 15 and 22, the mean times when T_b_ first decreased were 10:59±0:23, 10:14±0:27, 10:22±0:33 and 9:14±0:28 minutes which corresponded to mean T_b_ values of 38.49±0.07, 38.82±0.11, 38.75±0.14 and 38.72±0.18°C, respectively.

### (4) Effect of habitat on T_a_ and T_b_ daily rhythms

Mean daily T_a_ values were not significantly different between the two habitats (F_1,167_ = 0.188, P = 0.665). However, there were significant differences between habitats when day and night temperatures were specified in the model (Habitat: F_1,335_ = 0.939, p = 0.333; Day/night: F_1,335_ = 1131,p<0.001; Habitat * Day/night: F_1,335_ = 33.310, p<0.001) indicating that the flood plain was significantly hotter during the day and colder during the night than the woodland. Mean T_a_ values in the flood plain were 18.00±0.41°C during the day and 2.46±0.42°C during the night which compared with values of 15.34±0.40°C during the day and 4.35±0.37°C during the night in the woodland (Fig. 2D).

There was a significant effect of habitat on T_b_mesor and T_b_amplitude values. Values recorded for individuals from the flood plain were higher than those from the woodland (F_1,150_ = 10.23, p<0.01 and F_1,159_ = 81.58, p<0.001 respectively; Fig. 2A, 2B). However, there was no significant difference between T_b_acrophase values of individuals from the two habitats (F_1,127_ = 1.59, p = 0.210; Fig. 2C).

### (5) Effect of age and group size on T_b_ daily rhythms

**Figure 7:**
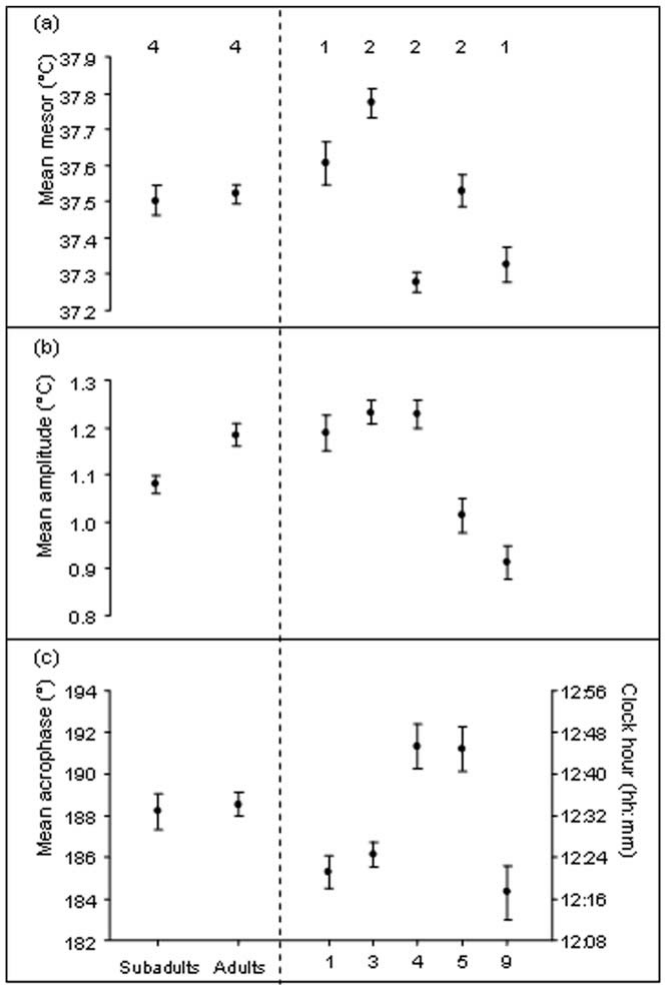
Mean ±SE values of the mesor, amplitude and acrophase shown per age class (subadults and adults) and for different group sizes (1, 3, 4, 5 and 9). The number of individuals in each category is indicated above the error bars. The parameters have been averaged for the level of individual (per category) and then for all weeks, hence SE is non-zero even when only data from one individual is presented.

There were significant interactions between age and body mass on T_b_mesor (F_1,111_ = 75.8, p<0.001 respectively). Older individuals decreased T_b_ with increasing mass whereas T_b_ was independent of body mass in younger animals. There was also a significant effect of group size on T_b_mesor with individuals from larger groups having lower T_b_mesor values than those from smaller groups (F_1,156_ = 18.70, p<0.001 respectively; Fig. 7A). There was a significant effect of group size (F_1,154_ = 22.29, p<0.001) and a significant interaction between age and body mass on T_b_amplitude (F_1,153_ = 9.22, p = 0.003). Individuals from larger group sizes had lower T_b_amplitude values and older animals decreased in T_b_amplitude with increasing mass whereas T_b_amplitude was independent of body mass in younger animals (Fig. 7B).There were significant interactions between age and body mass and between group size and body mass on T_b_acrophase (F_1,74_ = 44.26, p<0.001 and F_1,120_ = 36.25, p<0.001 respectively; Fig. 7C). Young animals which were large for their age tended to have T_b_acrophase values which occurred earlier in the day whereas larger adults had T_b_acrophase values which occurred later. Finally, T_b_acrophase values tended to occur later in the day as group size increased but was earliest for a group size of nine.

### (6) Effect of season, age and group size on the heterothermy index (HI)

Mean HI value across all individuals was 1.23±0.29°C and ranged from 0.68 to 2.32°C. While there were significant differences in HI values between individuals, there was no significant effect of ‘week’ (F_7,175_ = 22.91, p<0.001 and F_1,175_ = 1.15, p = 0.286). However, individuals from larger group sizes had lower HI values (least squares regression F_1,182_ = 20.33, p<0.001) and there was a significant interaction between age and group size on HI (F_1,180_ = 15.03, p<0.001); older animals decreased in HI with increasing group size whereas for young animals HI was independent of group size.

## Discussion

Living in hot arid environments can be stressful for small diurnal mammals since the availability of free water necessary to reduce body heat by evaporation is limited [49]. Consequently, evaporative cooling is often accompanied by behavioral and physiological mechanisms to dissipate heat such as the use of a thermal refuge or substrate [50] or heterothermy [13], [51]–[53]. In the current study, Cape ground squirrels were exposed to a wide seasonal and daily range of T_a_ and the T_b_mesor of all individuals increased significantly as the season progressed. This indicates that T_b_ values, including both maximal and minimal T_b_'s were higher on average when T_a_ values were higher. This will presumably serve to conserve their water and energy as a reduced T_a_-T_b_ temperature gradient minimizes the need to keep cool by evaporation [15], [54], [55]. In addition, acrophase values became earlier over the measurement period, indicating that activity periods also became earlier [28], [56]. Ground squirrels in general have labile T_b_'s [2], [5], [57]–[61], T_b_amplitudes of different species may vary by 4–5°C and be accompanied by bouts of torpor or hibernation. This compares with T_b_ amplitude values of up to 4.1°C in Arabian oryx (*Oryx leucoryx*) [51] and 2.6°C in Arabian sand gazelles (*Gazella subgutturosa marica*) [52]. We found no evidence of torpor and recorded daily variation in T_b_, of 5–6°C, which is greater than that noted in most other species and greater than noted by Wilson et al. (2010) [33] for Cape ground squirrels in a more mesic area (3.8°C amplitude); hence this probably reflects adaptation to an environment with high T_a_ values and large daily variations in T_a_.

### (3) Relationship between T_b_ daily rhythms, T_a_ and daylight

Peak ambient temperature (T_a_max) was the primary factor that explained both T_b_mean and T_b_amplitude, which suggests that this is the most thermally challenging period of the day. By comparison, sunrise provided the greatest explanatory power defining T_b_acrophase which may suggest that sunrise acted to temporally entrain the thermoregulatory system [62]. Indeed T_b_mean increased rapidly (4–5°C) post-emergence. The sensitivity of organisms to the timing of first light is exemplified by the fact that light ‘pollution’ during the dark phase can alter the seasonal acclimation of thermoregulatory, reproductive and immune systems of small mammals [63], [64]. Interestingly, increases in T_b_ during the first hour post-emergence were faster and greater earlier in the measurement period, indicating that animals gained thermal energy more rapidly during the winter. This indicates that as well as endogenous rhythms, mechanisms such as sun-basking might also be important in raising T_b_ [28], [31], [65], [66]. Whether or not squirrels preferentially orientate themselves to maximize heat uptake whilst basking, for example as in Raccoon dogs (*Nyctereutes procyonoides*) [67], remains unclear. By comparison, after initial increases, the time at which T_b_ stabilized in the mid-morning is likely to be indicative of another regulatory behavior: seeking shelter in burrows or in shade [31], [68]. This effect also became earlier as the season progressed (Fig. 7) suggesting that animals were using thermal refuges to offload heat earlier, allowing periodic bouts of foraging. There was also an indication that T_b_ tracked T_a_ (Fig. 4) highlighting the thermal lability of these animals. It is likely that Cape ground squirrels were allowing their T_b_ to vary to defend both water loss and energy expenditure as the greatest amplitudes of variation were noted during the winter. Alpine ibex (*Capra ibex ibex*) also show the greatest amplitude of variation of T_b_ during the winter which the authors suggested promoted a ‘thrifty’ use of body reserves [9]. By comparison, desert ungulates showed the greatest daily variation in T_b_ during the summer (2.6±0.8°C in Arabian sand gazelles and 4.1±1.7°C in Arabian oryx); this is the season that is most stressful for them when they benefit most by minimizing evaporative water loss [51], [52]. It is noteworthy that T_b_mean decreased just before evening immergence and remained steady once the squirrels were within their burrows. It seems that the major stimulus to enter burrows could be the prevention of a further decrease in T_b_ or an increase in energy expenditure due to increased thermoregulation, rather than other possible cues, such as light intensity.

### (4) Influence of habitat on T_b_ daily rhythms

As expected, T_a_ was more variable in the flood plain than in the woodland, with the former habitat exhibiting both colder nights and hotter days. Although the sample size was reduced because we were not able to capture many of the individuals that were implanted, the results obtained suggest that T_b_amplitude values were also greater in animals inhabiting the flood plain than the woodland. This may reflect a physiological strategy to minimize the T_a_-T_b_ temperature gradient and save on thermoregulatory costs [55]. There were also significant differences between T_b_mesor values of animals inhabiting the two habitats, with higher values recorded in those from the flood plain. This is interesting because T_a_mesor values did not differ between the two habitats. Therefore, the high T_a_ experienced during the day must have had a greater effect on the squirrels' physiology than the T_a_ experienced during the night in their burrows; moreover the flood plain was more thermally challenging than the woodland. Presumably squirrels are not exposed to the lowest T_a_ values during the night because they shelter in burrows, whereas they are exposed to high T_a_ values during the day even though they may use of temporary thermal refuges [68]. This corroborates our previous finding that T_a_max held the greatest explanatory power for and T_b_mesor.

The fact that variation in physiological characteristics occurred within a small geographical area suggests that Cape ground squirrels are able to regulate their T_b_ according to local environmental conditions. Similar patterns have been recorded in other small mammals albeit over different scales. Common spiny mouse (*Acomys cahirinus*) populations a mere 2–300 m apart on either side of a valley in the Mediterranean ecosystem exhibit a suite of physiological differences which include variations in their chronobiology [15], [69], as do populations of the broad-toothed field mouse (*Apodemus mystacinus*) from different sides of the African Great Rift valley [70], [71]. *A. cahirinus* inhabiting a xeric environment had later T_b_acrophase and greater T_b_amplitude values than those inhabiting a mesic cooler environment [15]. It was suggested that individuals from the former population allowed their T_b_ to vary considerably, rather than waste water by controlling T_b_ through evaporation or waste energy using endogenous heat sources, a strategy noted elsewhere [72]–[74]. Since no physical barrier exists between the two sites in the current study, one can assume that there is relatively high within-site fidelity [40].

### (5) Effects of age and group size on T_b_ variation

Across taxa, younger animals generally have less prominent T_b_ daily rhythms than older animals, in part because T_b_ daily rhythms need time to mature [75], [76]. Larger animals also tend to have smaller T_b_amplitude values as a presumed consequence of their greater thermal inertia and reduced susceptibility to changes in food availability [76], [77]. Although our results must be interpreted with caution because of the small sample sizes, these relationships are corroborated as a negative correlation was noted between T_b_mean and body mass in older but not in younger animals. In our case, heavy young animals also tended to have earlier T_b_acrophase values, indicating earlier activity periods in these individuals. If emergence times are driven by thermoregulatory constraints, it is possible that older individuals and those large for their age may emerge earlier because of their lower surface area to volume ratios and greater thermal capacities. An alternative explanation might be that larger animals might simply have more fat reserves, allowing them to emerge earlier and expend more energy on thermoregulation.

The fact that T_b_mesor values decreased with increasing group size suggests that squirrels were expending less energy on thermoregulation in larger groups. Previous studies have suggested that aggregation/huddling behavior can significantly reduce thermoregulatory costs [17], [78] and daily averaged energy expenditure [79] in some groups of small mammals. For example, T_b_ values were found to be lower in large groups of roosting bats *Noctilio albiventris* [80]. It was suggested that individual bats in larger groups might be less prone to predation and hence could benefit by lowering their T_b_'s further than those within smaller groups. In contrast, for two species of African mole-rat (*Cryptomys hottentotus natalensis* and *Fukomys damarensis*), individuals in experimentally increased group sizes had greater T_b_ values [78]. In this case a crowded burrow which is thermally buffered might make it difficult to cool down and consequently T_b_ values are greater. Because Cape ground squirrels forage during the day as a spaced group [35], any thermoregulatory benefits of group size would presumably occur during the night [68] and hence a larger group size could facilitate a lower and more stable T_b_.

Finally, both T_b_amplitude and HI were negatively associated with group size and older animals had lower HI values in larger group sizes whereas younger animals did not. This is also consistent with our predictions that individuals in larger groups benefit by being thermally buffered and that older animals are better at regulating their T_b_. In this instance, both metrics (T_b_amplitude and HI) appear to provide similar results, i.e. that there are significant effects of age and group size on T_b_ variation. Overall, these data confirm that the thermal physiology of Cape ground squirrels is sensitive to changes both in the abiotic and biotic environment. Many factors are observed to affect their T_b_, which can be modified, enabling them to survive in arid, hostile environments.
