# Correction: Seasonal Patterns of Body Temperature Daily Rhythms in Group-Living Cape Ground Squirrels *Xerus inauris*

**DOI:** 10.1371/annotation/65754bea-b508-431f-acf8-6c2a26602f28

**Published:** 2012-08-08

**Authors:** Michael Scantlebury, Marine Danek-Gontard, Philip W. Bateman, Nigel C. Bennett, Mary Beth Manjerovic, Kenneth E. Joubert, Jane M. Waterman

The fifth author's name was spelled incorrectly. The correct name is: Mary Beth Manjerovic. The correct citation is: Scantlebury M, Danek-Gontard M, Bateman PW, Bennett NC, Manjerovic MB, et al. (2012) Seasonal Patterns of Body Temperature Daily Rhythms in Group-Living Cape Ground Squirrels Xerus inauris. PLoS ONE 7(4): e36053. doi:10.1371/journal.pone.0036053 

